# Protective Effects of Five Structurally Diverse Flavonoid Subgroups against Chronic Alcohol-Induced Hepatic Damage in a Mouse Model

**DOI:** 10.3390/nu10111754

**Published:** 2018-11-14

**Authors:** Liang Zhao, Nanhai Zhang, Dong Yang, Mengyan Yang, Xiaoxuan Guo, Jiguo He, Wei Wu, Baoping Ji, Qian Cheng, Feng Zhou

**Affiliations:** 1 Beijing Advanced Innovation Center for Food Nutrition and Human Health, Beijing Key Laboratory of Functional Food from Plant Resources, College of Food Science & Nutritional Engineering, China Agricultural University, 17 Tsinghua East Road, Beijing 100083, China; liangzhao@cau.edu.cn (L.Z.); nanhaizhang@cau.edu.cn (N.Z.); dyang@cau.edu.cn (D.Y.); hejiguo0870@sina.com (J.H.); jbp@cau.edu.cn (B.J.); 2 College of Information and Electrical Engineering, China Agricultural University, 17 Tsinghua East Road, Beijing 100083, China; myyang@cau.edu.cn; 3 Institute of Quality Standard and Testing Technology for Agro-Products, Chinese Academy of Agricultural Sciences, 12 Zhongguancun South Street, Beijing 100081, China; guoxiaoxuan@caas.cn; 4 College of Engineering, China Agricultural University, 17 Tsinghua East Road, Beijing 100083, China; wuwei70@126.com; 5 Hubei Provincial Key Laboratory of Yeast Function, Angel Yeast Co. Ltd., 24 Chengdong Road, Yichang 443003, China

**Keywords:** apigenin, quercetin, naringenin, (-)-epigallocatechin gallate, genistein, liver injury

## Abstract

Alcoholic liver disease (ALD) has become one of the major global health problems, with augmented morbidity and mortality. Evidence indicates that flavonoids can reduce the risk of ALD owing to their biological properties. However, the effect of structurally different flavonoid subclasses on alleviating alcohol-induced liver damage in a same model has never been studied. In this study, mice were supplemented with five kinds of flavonoid subgroups, apigenin (flavone), quercetin (flavonol), naringenin (flavanone), (-)-epigallocatechin gallate (flavanol), and genistein (isoflavone), in the same dose (0.3 mmol kg^−1^ body weight) and then given 50% alcohol by gastric perfusion for five consecutive weeks. The results demonstrated that genistein and naringenin had greater benefits in terms of mitigating fibrosis and apoptosis, respectively, in the liver. Lipid deposition, partial inflammatory-related factors (nuclear factor kappa B p65, cyclooxygenase-2, and interleukin-6 levels), and hepatic histopathological alterations were similarly attenuated by five kinds of flavonoids. All the flavonoids also showed different degrees of influence on protecting against alcoholic liver injury on other aspects, such as serum biochemistry makers, hepatic lipid accumulation, lipid peroxidation, antioxidant capacities, and inflammation.

## 1. Introduction

Per capita alcohol consumption has risen rapidly in many countries, and excessive alcohol consumption, the third largest risk for disease in the world, has increased morbidity and mortality from alcoholic liver disease (ALD), which has become one of the most critical public health issues [1,2,3]. Meanwhile, excessive alcohol consumption can aggravate economic burdens on society, and leads to about 3 million deaths annually [1,4]. ALD usually elicits a series of histological phenotypes of alcohol-related liver injury, including the earliest response of fatty liver or simple steatosis (an accumulation of fat in hepatocytes), steatohepatitis (steatosis with superimposed inflammatory infiltrate), and ultimate progression to fibrosis, cirrhosis, and hepatocellular carcinoma [5]. These stages were expressed as deposition of lipid droplets, total triglyceride (TG), and total cholesterol (TC) in serum and liver, reduction in antioxidant enzymatic activities in serum and liver, overproduction of hepatic lipid peroxidation product, pro-inflammatory cytokines, apoptotic protein, mitochondrial dysfunction, etc. [6,7]. At present, there is increasing interest in seeking ideal and safe components as alternatives, especially from plant sources, for the amelioration of ALD in the presence of some medicines with side effects [8].

Flavonoids, the largest class of naturally polyphenolic compounds and a group of biologically active secondary metabolites produced from plants, are ubiquitously distributed and accumulate in relatively high concentrations in all foods of plant origin [9,10]. Flavonoids, possessing a C_6_-C_3_-C_6_ structure (comprising two aromatic rings connected by three carbons), are usually distinguished into six major subgroups with different structures on account of the diversely central C-ring including flavones, flavonols, flavanones, flavanols, isoflavones, and anthocyanidins [9]. Increasing evidence indicates that flavonoids exert positive effects against lipid metabolism, oxidative stress, inflammatory, insulin resistance, apoptosis, cellular proliferation, tumor, and on hippocampal brain-derived neurotrophic factor, thereby benefiting non-alcohol-/alcohol-induced liver disease, type II diabetes, cardiovascular disease, cancer, memory, and cognition [6,11,12]. Due to their multiple functions, flavonoids are considered as safe protective agents in the prevention or treatment of hepatic injury.

**Figure 1:**
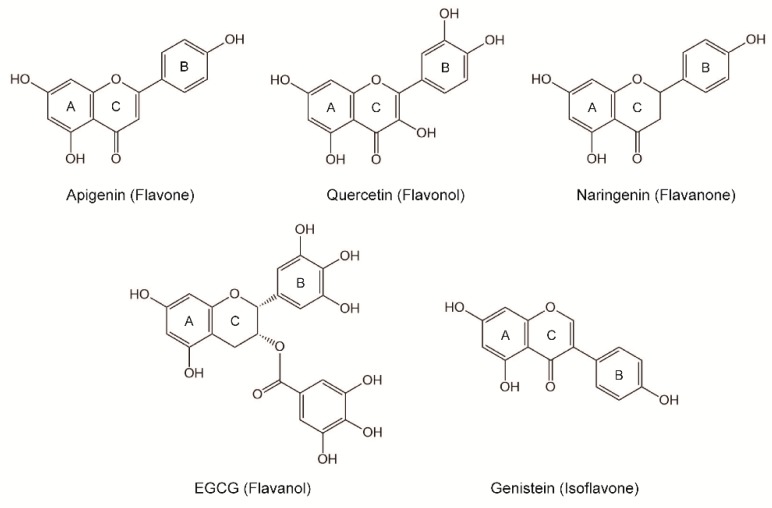
Structures of the flavonoids considered in the current study.

Apigenin, quercetin, naringenin, (-)-epigallocatechin gallate (EGCG), and genistein are the main representatives of flavones, flavonols, flavanones, flavanols, and isoflavones, respectively, and their structures are displayed in Figure 1 [10]. Several reports investigated how these five flavonoids alleviate the liver injury resulting from alcohol consumption [13,14,15,16]. However, experiments in these studies were conducted in different animal models with different doses of flavonoids, which makes it difficult to compare the structurally variant effects on alcohol-induced liver damage between different flavonoid subclasses. Moreover, the potential mechanism of the protective effects of different flavonoids on liver injury induced by chronic alcohol intake remains unclear. Therefore, a comparison of the hepatoprotective effects of different flavonoids at the same level is needed to provide information for exploiting new naturally occurring functional components that could be added to various foods to prevent or reduce the risk of ALD.

In the present study, we aimed to investigate the differences in the effects of purified flavone (apigenin), flavonol (quercetin), flavanone (naringenin), flavanol (EGCG), and isoflavone (genistein) on liver injury by alcohol in mice. The underlying mechanisms of the protective effects (antioxidant, anti-inflammatory, anti-fibrotic, and anti-apoptotic activities) were also investigated. Specifically, the levels of serum transaminases, serum and hepatic lipid profiles, hepatic lipid peroxidation products, antioxidant capacities, inflammatory mediators, pro-inflammatory cytokines, profibrotic cytokine, apoptotic protein, and liver histopathology were determined.

## 2. Materials and Methods

### 2.1. Chemicals and Reagents

Apigenin (98%), quercetin (98%), naringenin (98%), EGCG (98%), and genistein (99%) were purchased from Nanjing Jingzhu Biotechnology Co., Ltd. (Nanjing, China). Commercial assay kits for aspartate aminotransferase (AST), ALT, TG, TC, high-density lipoprotein cholesterol (HDL-C), and LDL-C were purchased from Biosino Bio-Technology and Science Inc. (Beijing, China). Detection kits including MDA, catalase (CAT), superoxide dismutase (SOD), glutathione (GSH), and glutathione peroxidase (GSH-Px) were obtained from Nanjing Jiancheng Bioengineering Institute (Nanjing, China). Enzyme-linked immunosorbent assay (ELISA) kits of heme oxygenase-1 (HO-1), NF-*κ*B p65, transforming growth factor *β*1 (TGF-*β*1), cyclooxygenase-2 (COX-2), monocyte chemoattractant protein-1 (MCP-1), inducible nitric oxide synthase (iNOS), TNF-*α*, IL-6, and caspase-3 were obtained from Keyingmei Biotechnology and Science Inc. (Beijing, China). A bicinchoninic acid assay (BCA) kit was purchased from Beyotime Biotechnology Inc. (Beijing, China). Other chemicals and reagents, such as alcohol, were all of analytical grade.

### 2.2. Animals and Experimental Design

Seventy male ICR mice, 20–22 g, were provided by Beijing Vital River Laboratory Animal Technology Co., Ltd. (Beijing, China) [Certificate SCXK (Beijing) 2012-0001]. For one week of acclimation, the mice were maintained in a specific environment under a 12 h light/dark cycle at 22 ± 2 °C ambient temperature and 55 ± 5% relative humidity. All experimental procedures in this study involving animals were approved by the Ethics Committee of the Beijing Key Laboratory of Functional Food from Plant Resources (Permit number: A330-10) and strictly complied with the guidelines for the care and use of laboratory animals of the National Institutes of Health.

The animals were randomly divided into seven groups (consisting of 10 mice per group), including a normal control group (NG), a model group (MG), and flavonoid groups (treatment with quercetin, apigenin, naringenin, epigallocatechin gallate, and genistein, respectively), and were fed with water and a standard pellet diet ad libitum. The NG was given 0.5% sodium carboxymethyl cellulose solution (CMC-Na) by an oral route. The MG was administered 10 mL kg^−1^ body weight of 50% alcohol by oral gavage after 1 h of 0.5% CMC-Na for five weeks. Mice in the flavonoid groups were supplied orally with five kinds of different flavonoids (suspended in 0.5% CMC-Na) at an equimolar concentration of 0.3 mmol kg^−1^ body weight for each group and then received 50% alcohol 1 h later every day. The total experimental duration was five weeks. Mice were weighed every three days and the gastric infusion volume per day was regulated with their body weight. Finally, all the animals were weighed and sacrificed after a 12-h fast. Blood samples were collected from the retro-orbital venous plexus by capillary tube and liver tissues were dissected out. The fresh liver was washed and weighed to calculate the liver index [liver index (%) = liver weight/final body weight × 100%]. Then one part of the liver tissues was soaked in liquid nitrogen or a formaldehyde solution for hepatic histopathological observation. The other hepatic sections were homogenized and immediately stored at −80 °C for further assays.

### 2.3. Determination of Serum Biochemical Parameters

Blood samples were stored at 4 °C to clot for 6 h and then serum was obtained by centrifugation at 4000 *g* for 15 min at 4 °C, after which serum biochemical parameters including AST, ALT, HDL-C, LDL-C, TG, and TC were measured on an Alcyon 300 automatic analyzer (Abbott, Chicago, IL, USA) using the corresponding detection kits according to the instruction manuals.

### 2.4. Determination of Hepatic Biochemical Parameters

The total protein concentrations in the liver homogenate were determined by a BCA kit. The lipids were extracted from the liver homogenate following the method of Wang [8]. Hepatic lipid profiles (TG and TC levels) were measured using the same method used for serum TG and TC levels. Then the lipid peroxidation product (MDA level) and antioxidant markers (SOD, CAT, GSH, and GSH-Px activities) in liver tissues were assayed on an Alcyon 300 automatic analyzer by using the corresponding kits following the manufacturer’s instructions. ELISA kits were used to determine the levels of HO-1, NF-*κ*B p65, COX-2, MCP-1, iNOS, TNF-*α*, IL-6, TGF-*β*1, and caspase-3. Values were normalized to the total protein concentration.

### 2.5. Liver Histopathology

Hematoxylin and eosin (H&E) and oil red O staining were utilized for liver histopathology. Upon H&E staining, liver specimens were fixed in 10% neutral buffered formaldehyde solution, embedded in paraffin, sliced with a microtome, and stained with H&E. The process of oil red O staining consisted of freezing the specimens in liquid nitrogen, thinly sectioning the specimens, and staining with oil red O solution dissolved in isopropanol to a concentration of 5 mg/mL. A light microscope (BA-9000L, Osaka, Japan) was used to examine the histopathological changes of hepatic sections.

### 2.6. Statistical Analysis

Data were tested for normality of distribution and equality of variance using the Kolmogorov–Smirnov and Levene tests, respectively. If normally distributed and homogeneous, data were analyzed by one-way analysis of variance (ANOVA) followed by Duncan’s test to assess the statistical significance of the differences between groups. In the case where data were not normally distributed or nonhomogeneous, data were analyzed using a Kruskal‒Wallis test followed by Dunn’s multiple range post-test. Data were expressed as means ± standard deviation (SD) and differences when *p* < 0.05 were considered significant. All statistical analyses were performed by Prism 7.0 (GraphPad Software, San Diego, CA, USA).

## 3. Results

### 3.1. Effect of Five Kinds of Flavonoids on Food Intake, Body Weight, Liver Weight, and Liver Index in Mice

**Figure 2:**
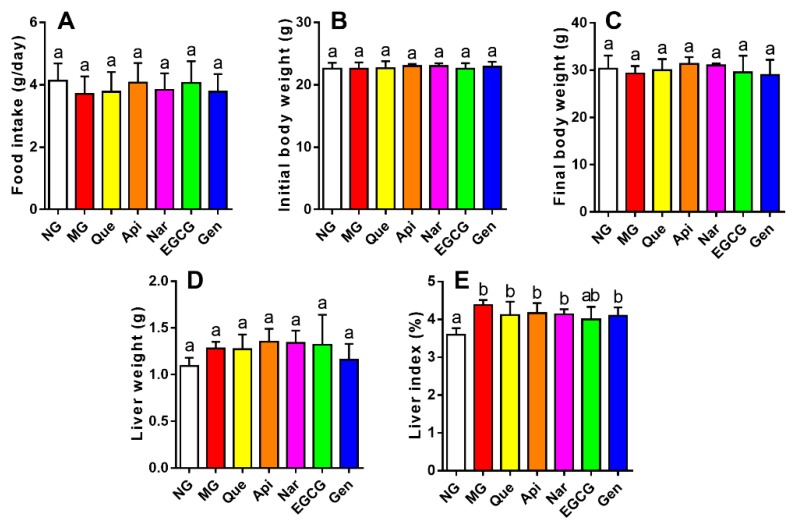
Effect of five kinds of flavonoids on food intake, body weight, liver weight, and liver index in mice. (**A**) Food intake; (**B**) initial body weight; (**C**) final body weight; (**D**) liver weight; (**E**) liver index. Values are expressed as the mean ± SD (*n* = 10). Labeled means without a common letter differ (*p* < 0.05). NG, normal group; MG, model group; Que, quercetin; Api, apigenin; Nar, naringenin; EGCG, epigallocatechin gallate; Gen, genistein.

As displayed in Figure 2A–D, there were no obvious changes in food intake, initial body weight, final body weight, and liver weight among seven groups. The liver index of mice in the MG was higher than NG (*p* < 0.05, Figure 2E), while no significant differences were found between these flavonoid groups and the MG, indicating that all the flavonoids showed no significant effect on alleviating the liver index.

### 3.2. Effect of Five Kinds of Flavonoids on Serum Biochemical Markers

Table 1 presents the variation of AST, ALT, HDL-C, LDL-C, TG, and TC in the serum of control and treated mice. The activities of AST and ALT, as well as the levels of LDL-C, TG, and TC showed a remarkable elevation after alcohol consumption (vs. NG, *p* < 0.05), with a simultaneous decrease in HDL-C level (vs. NG, *p* < 0.05). Administration of flavonoids not only alleviated AST and ALT activities and LDL-C level, but also significantly enhanced the HDL-C level relative to that in the MG (*p* < 0.05). However, among the five flavonoid groups, the naringenin and apigenin groups showed lower serum ALT activity; mice treated with genistein showed less AST activity in serum; apigenin had a more efficient effect on decreasing the LDL-C level in serum; the EGCG group achieved a higher HDL-C level in serum than the other flavonoid groups. Moreover, compared with the MG, all the flavonoids significantly reduced the levels of TG and TC in serum with no obvious difference (*p* < 0.05). Therefore, these results indicated that pretreatment with all the flavonoids could play a role in elevating hepatic function and preventing dyslipidemia in serum to varying degrees.

### 3.3. Effect of Five Kinds of Flavonoids on Hepatic Lipid Profiles

**Figure 3:**
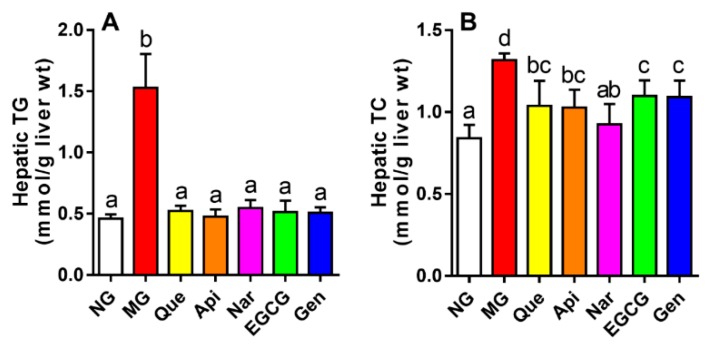
Effect of five kinds of flavonoids on hepatic lipid profiles: (**A**) hepatic total triglyceride level; (**B**) hepatic total cholesterol level. Values are expressed as the mean ± SD (*n* = 10). Labeled means without a common letter differ (*p* < 0.05). NG, normal group; MG, model group; Que, quercetin; Api, apigenin; Nar, naringenin; EGCG, epigallocatechin gallate; Gen, genistein; TG, total triglyceride level; TC, total cholesterol level.

Compared to the NG, the results revealed considerable upregulation of hepatic TG and TC contents after alcohol consumption by 3.3- and 1.6-fold, respectively (*p* < 0.05, Figure 3). After treatment with these five flavonoids, the hepatic TG concentration markedly declined (vs. MG, *p* < 0.05) to the equivalent level with the NG, whereas no obvious change was found between the flavonoid groups. In addition, flavonoid supplementation to alcohol-fed mice also markedly reduced the hepatic TC level (vs. MG, *p* < 0.05), especially in the naringenin group, in which the level of TC in the liver was lower than in the other flavonoid groups.

### 3.4. Effect of Five Kinds of Flavonoids on Hepatic Lipid Peroxidation and Oxidative Stress

**Figure 4:**
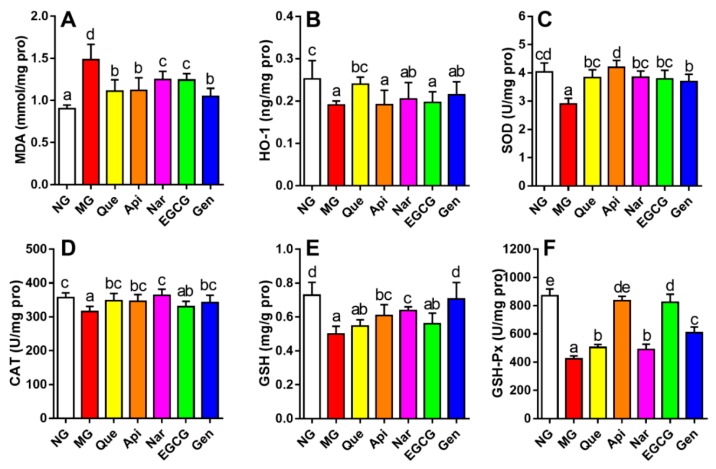
Effect of five kinds of flavonoids on hepatic lipid peroxidation and oxidative stress: (**A**) malondialdehyde level; (**B**) heme oxygenase 1 level; (**C**) superoxide dismutase activity; (**D**) catalase activity; (**E**) glutathione activity; (**F**) glutathione peroxidase activity. Values are expressed as the mean ± SD (*n* = 10). Labeled means without a common letter differ (*p* < 0.05). NG, normal group; MG, model group; Que, quercetin; Api, apigenin; Nar, naringenin; EGCG, epigallocatechin gallate; Gen, genistein.

As shown in Figure 4, the hepatic MDA level in the mice induced by alcohol consumption was remarkably augmented and the activities of HO-1, CAT, SOD, GSH, and GSH-Px were apparently inhibited as compared with those in the NG (*p* < 0.05). The production of hepatic lipid peroxidation product and oxidative stress was inhibited to varying extents resulting from the treatment with flavonoids relative to the MG. Compared to the MG, quercetin, apigenin, and genistein had a more beneficial effect on decreasing the MDA level; the activities of SOD and CAT were raised more in the apigenin and naringenin groups, respectively; administration of quercetin produced a higher HO-1 level; genistein enhanced the activity of GSH; and apigenin elevated the activity of GSH-Px more than the other flavonoids.

### 3.5. Effect of Five Kinds of Flavonoids on Hepatic Inflammatory Stress

**Figure 5:**
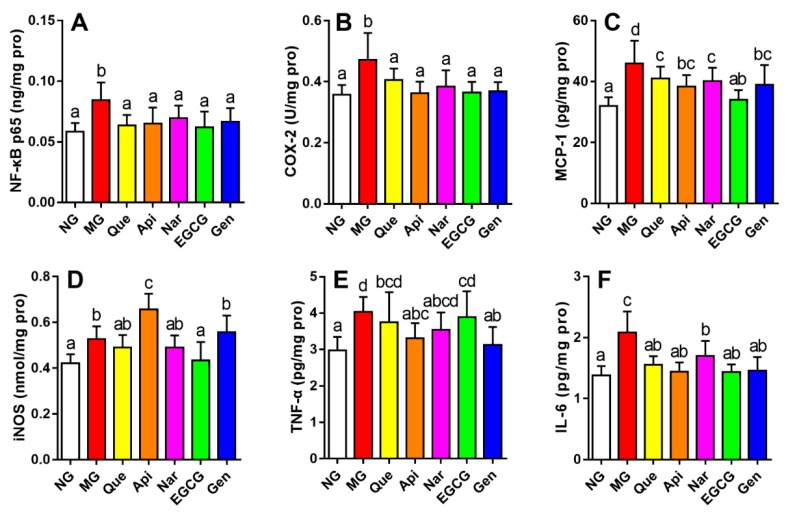
Effect of five kinds of flavonoids on hepatic inflammatory stress: (**A**) nuclear factor kappa B p65 level; (**B**) cyclooxygenase 2 level; (**C**) monocyte chemoattractant protein 1 level; (**D**) inducible nitric oxide synthase level; (**E**) tumor necrosis factor *α* level; (**F**) interleukin-6 level. Values are expressed as the mean ± SD (*n* = 10). Labeled means without a common letter differ (*p* < 0.05). NG, normal group; MG, model group; Que, quercetin; Api, apigenin; Nar, naringenin; EGCG, epigallocatechin gallate; Gen, genistein.

The alterations of inflammatory mediators (NF-*κ*B p65, COX-2, MCP-1, and iNOS levels) and pro-inflammatory cytokines (TNF-*α*, and IL-6 levels) in mice liver tissue were presented in Figure 5 to evaluate the effect of flavonoids on hepatic inflammatory stress. Mice induced by alcohol consumption were considerably higher on these indices than those in NG (*p* < 0.05). Treatment with flavonoids could prevent an elevation in all indicators to a different extent. Compared with the MG, EGCG showed a significant effect, suppressing the levels of MCP-1 and iNOS (*p* < 0.05); genistein markedly decreased the level of TNF-*α* (*p* < 0.05). In addition, all the flavonoids caused a decrease in the levels of NF-*κ*B p65, COX-2, and IL-6 with respect to the MG (*p* < 0.05), while the results showed no statistical differences among the flavonoid groups.

### 3.6. Effect of Five Kinds of Flavonoids on Hepatic Fibrosis and Apoptosis

**Figure 6:**
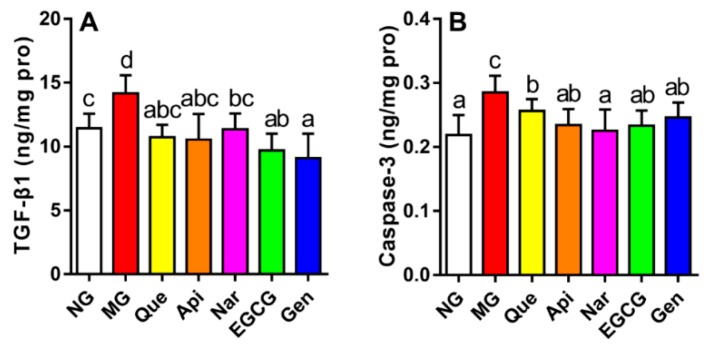
Effect of five kinds of flavonoids on transforming growth factor *β*1 (**A**) and caspase-3 (**B**) levels. Values are expressed as the mean ± SD (*n* = 10). Labeled means without a common letter differ (*p* < 0.05). NG, normal group; MG, model group; Que, quercetin; Api, apigenin; Nar, naringenin; EGCG, epigallocatechin gallate; Gen, genistein.

Alterations in the levels of TGF-*β*1 and caspase-3 were examined in the livers of mice treated with these five flavonoids to assess the effect of flavonoids against hepatocellular fibrosis and apoptosis induced by alcohol intake respectively (Figure 6). Compared to the NG, we observed an expected increase in TGF-*β*1 and caspase-3 levels in the MG (*p* < 0.05). After the administration of flavonoids, the growth of TGF-*β*1 and caspase-3 levels was markedly inhibited in comparison with the MG (*p* < 0.05). Additionally, genistein significantly reduced the TGF-*β*1 level as compared with naringenin (9.11 ± 1.895 vs. 11.35 ± 1.239 ng/mg pro, *p* < 0.05), whereas the other flavonoids showed no apparent effect on decreasing the TGF-*β*1 level relative to genistein and naringenin. Similarly, the naringenin group achieved a lower caspase-3 level than the quercetin group (0.23 ± 0.034 vs. 0.26 ± 0.018 ng/mg pro, *p* < 0.05), while the other flavonoids produced no obvious improvement in terms of decreasing the caspase-3 level compared to naringenin and quercetin.

### 3.7. Liver Histopathological Analysis

**Figure 7:**
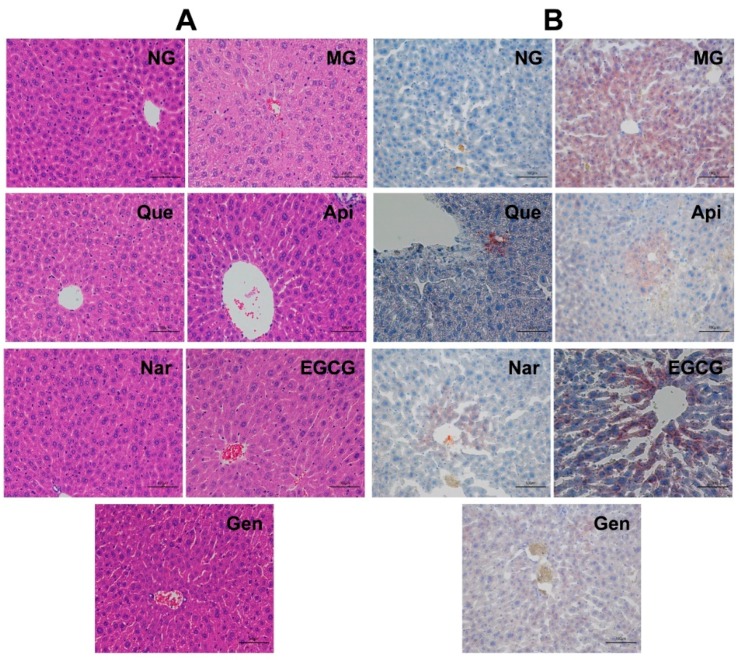
Histopathological detection of livers. Typical photomicrographs of liver sections at a magnification of 200× were stained with hematoxylin and eosin (**A**) and oil red O (**B**). All the bars represent 100 μm. NG, normal group; MG, model group; Que, quercetin; Api, apigenin; Nar, naringenin; EGCG, epigallocatechin gallate; Gen, genistein.

H&E and oil red O staining were used to visualize the histopathological differences between liver tissue sections from each group (Figure 7). The results of H&E staining assay illustrated that the liver section of the NG mice showed no histological alterations and exhibited a uniform hepatocellular architecture with portal vein, radiating hepatic cords, normal hepatic sinusoid, well-preserved cytoplasm, clearly visible nucleus and nucleolus (Figure 7A). On the other hand, chronic alcohol feeding for five weeks created several degrees of lesions with fatty vacuoles, edema, cell swelling, and disappearance of hepatic sinusoid. The administration of flavonoids markedly mitigated these hepatocyte alterations induced by chronic alcohol supplementation, whereas no pronounced differences were shown between hepatocyte sections in flavonoid groups.

Massive hepatic fat accumulation was observed in the liver section of MG mice according to oil red O staining (Figure 7B). In contrast, flavonoid treatment noticeably reduced the level of lipid deposition relative to MG, and their liver tissues were comparable to those of the NG. There are also no obvious changes between the flavonoid groups, which was in accordance with the results of hepatic TG. These results suggested that these five flavonoids could significantly ameliorate alcohol-induced histopathological changes.

## 4. Discussion

ALD has become one of the major causes of death worldwide as the number of individuals drinking long term has increased [14,17]. It was thought that the progression of ALD was mainly contributed to oxidative stress, production of cytokines by adipocytes and inflammatory cells, and apoptosis [2]. Flavonoids had been reported to display antioxidant, anti-inflammatory, anti-fibrosis, and anti-apoptosis properties, which meant they had the capacity to counteract ALD [18]. In this study, five flavonoid subgroups (apigenin, quercetin, naringenin, EGCG, and genistein) were chosen to compare their hepatoprotective effect under chronic alcohol consumption in the same gavage dose and animal model.

In this work, although the liver weight index was significantly increased in the MG (vs. NG, *p* < 0.05, Figure 2), there were no apparent changes in flavonoid groups compared to MG. Usually, serum biochemical makers are the earliest sign of liver injury [19]. The liver membranes would rupture due to alcohol intake, leading to the release of aminotransferase into the serum and then the elevation of serum AST and ALT activities [19,20]. Previous reports showed that genistein, naringenin, quercetin, and EGCG obviously inhibited the activities of AST and ALT in serum induced by alcohol [21,22,23,24]. In our study, we observed remarkably enhanced activities of AST and ALT (vs. NG, *p* < 0.05, Table 1) after administration with alcohol for five weeks. The ALT activity was more attenuated by the treatment with apigenin and naringenin, whereas genistein was more effective at lowering the AST activity, mitigating alcohol-induced liver damage. Moreover, supplementation of flavonoids could alleviate the histopathological alterations with fatty vacuolization in the liver (Figure 7A).

Adenosine monophosphate-activated protein kinase (AMPK) is known to block anabolic pathways, but to stimulate catabolism [25]. Accordingly, alcohol intake could decrease AMPK expression, which activated lipogenic enzymes through upregulating the level of sterol regulatory element-binding protein (SREBP) and downregulating the level of peroxisome proliferator- activated receptor *α* (PPAR*α*), thereby inducing lipid accumulation, suppressing mitochondrial fatty acid oxidation or export, and ultimately leading to hepatic steatosis [5,25,26]. This action was driven by the overstorage of energy in hepatocytes produced by NADH with the alcohol metabolism [27]. It is reported that AMPK could increase the expression of SREBP-1 and SREBP-2 involved in TG synthesis and cholesterol homeostasis, and inhibit the expression of carnitine palmitoyltransferase-1 and microsomal triglyceride transfer protein involved in fatty acid oxidation or transport [5,25]. Wang et al. indicated that treatment with apigenin (300 mg kg^−1^ per day for 30 days) could regulate lipid synthesis via increasing the expression of PPAR*α* and decreasing the expression of SREBP-1c [13]. It had also been reported that naringenin (50 mg kg^−1^ per day for 30 days) could reduce TC and TG levels in serum and liver, and EGCG (100 mg kg^−1^ per day for 30 days) could apparently alter the levels of TG, LDL-C, and HDL-C to prevent the accumulation of fat [28,29]. In fact, the present results demonstrated that all the flavonoids similarly alleviated the serum and hepatic TG level (vs. MG, *p* < 0.05, Table 1 and Figure 3A), and naringenin was more effective at mitigating the hepatic TC level (Figure 3B). Meanwhile, apigenin, naringenin, and genistein had a more beneficial effect in terms of ameliorating the serum TC level (Table 1). Additionally, the mice supplemented with EGCG and apigenin gained a higher HDL-C level and lower serum LDL-C level, respectively (Table 1), which was in agreement with the previous results. Similarly, the lipid deposition in mice induced by alcohol could be directly alleviated by all the flavonoids (Figure 7B). The results might be explained by activating the AMPK pathway to suppressing the accumulation of fat and cholesterol homeostasis at different levels for different kinds of flavonoids.

Oxidative stress has been reported to play a vital role in the development of ALD [19]. In the alcoholic metabolism, free radical intermediates like reactive oxygen species (ROS) are generated by the endoplasmic reticulum, Kupffer cells, and mitochondria, which could cause oxidant imbalance, resulting in oxidative stress and hepatocellular damage, as well as lipid peroxidation [26,30]. Evidence revealed that alcohol consumption could increase hepatic MDA content, promoting lipid peroxidation and then failing to prevent excessive free radical intermediates, thereby enhancing oxidative stress [31]. In the present study, quercetin, apigenin, and genistein obtained less MDA content relative to other flavonoids (*p* < 0.05, Figure 4A). HO-1 could not only inhibit the production of ROS and downstream antioxidant enzymes activities through counteracting hepatic cytochrome P450 2E1 (CYP2E1) in the endoplasmic reticulum to protect against oxidative stress, but also suppress apoptosis and cellular inflammation [3,6,26]. A recent report suggested that genistein or apigenin pretreatment could strengthen hepatic antioxidant ability, which contributed to inhibiting the production of CYP2E1-mediated reactive oxygen species, then downregulating the oxidative stress, and finally attenuating alcoholic liver injury [13,21]. Likewise, quercetin and EGCG supplementation could improve the inhibited antioxidant enzyme activity or block the MDA contents in liver resulting from chronic alcohol intake [24,32]. Our data denoted that, although quercetin was more effective at enhancing the HO-1 level (Figure 4B), apigenin, naringenin, and genistein had a more efficient effect in terms of increasing the activities of SOD, CAT, GSH, and GSH-Px (Figure 4C–F), in agreement with former results. Therefore, different kinds of flavonoids could ameliorate oxidative stress to a varying degree via inhibiting the production of MDA and augmenting the antioxidant enzymatic activities. Conversely, compared to MG, no significant effects were observed in HO-1 activity in the apigenin, naringenin, EGCG, and genistein groups, CAT activity in the EGCG group, and GSH activity in the quercetin and EGCG groups. Probably due to the number and position of hydroxyl groups on the B-ring in different flavonoids, variation in the antioxidant capacities and TG inhibitory effect could occur [33].

Inflammation response is a pathological event associated with the ALD process [30]. When activation of NF-*κ*B occurred in the hepatocellular nucleus, some genes was rapidly induced to inflammation and apoptosis, resulting in the expression of inflammatory mediators (such as COX-2, MCP-1, and iNOS) and facilitating the production of pro-inflammatory cytokines (such as TNF-*α* and IL-6), which could lead to the production of ROS [6,8,27]. Accordingly, NF-*κ*B is well known as a major mediator of cellular inflammation and necrosis [34]. It was argued in previous studies that genistein could reduce the levels of inflammatory factors via suppressing the NF-*κ*B level, as well as EGCG [21,24]. In this study, all the flavonoids could downregulate the levels of NF-*κ*B p65, COX-2 and IL-6 (*p* < 0.05, Figure 5A,B,F). Similarly, EGCG could provoke a beneficial effect by attenuating MCP-1 and iNOS levels, and the genistein group showed a higher TNF-*α* level than other flavonoids (Figure 5C,D). Nevertheless, consumption of quercetin, naringenin, genistein, and apigenin had no considerable effects on the iNOS level, and supplementation of quercetin, naringenin, and EGCG exerted no marked function on the TNF-*α* level. Probably, these flavonoids modulated the inflammation response through the NF-*κ*B pathway with a different influence in downstream proteins.

Hepatic fibrosis mediated by inflammation is a form of alcoholic liver disease that is mainly attributed to profibrotic cytokines [3]. Animal experiments have confirmed that TGF-*β*1 is essential to liver fibrosis, thereby unquestionably becoming one of the most powerful cytokines promoting liver fibrosis among numerous inflammatory mediators [3,35]. In our study, administration of flavonoids could apparently reduce the enhanced TGF-*β*1 level induced by alcohol consumption (*p* < 0.05, Figure 6A). Genistein, especially, was more effective at ameliorating fibrosis of liver cells than other flavonoids via inhibiting the TGF-*β*1 level. In the same way, Huang et al. reported that genistein treatment (0.5–2 mg kg^−1^ each day for 24 weeks) could suppress hepatic fibrosis by decreasing the production of TGF-*β*1 and Smad 3 [21].

Hepatocyte apoptosis is recognized as a vital pathologic feature of ALD [36]. Caspases drive the critical process of apoptosis, among which caspase-3 is widely studied and has become the main driver of apoptosis due to the presence of many cellular targets [37,38]. Evidence also demonstrated that functional components, such as genistein, puerarin, and zeaxanthin dipalmitate, could protect against alcohol-induced hepatocellular apoptosis via preventing an increase in the level of caspase-3 [6,39]. Our results supported this theory since the hepatocyte apoptosis emerged in mice subjected to chronic alcohol administration, which was remarkably inhibited by treatment with all the flavonoids (Figure 6B). In addition, naringenin had a more efficient effect in terms of attenuating the hepatocyte apoptosis than other groups in view of its lowest caspase-3 level, which suggested that naringenin may to some extent alleviate hepatic apoptosis.

## 5. Conclusions

**Figure 8:**
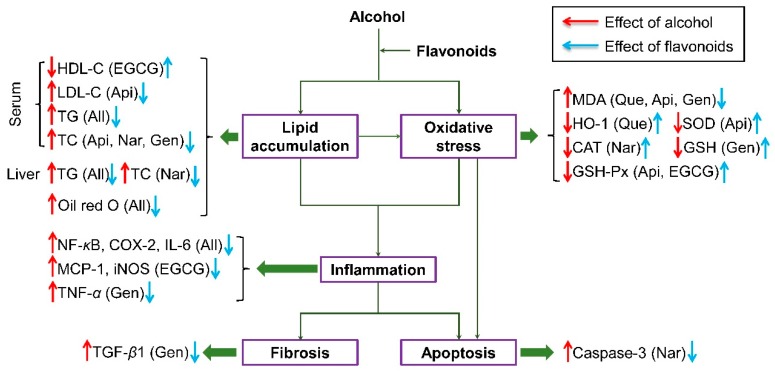
Schematic diagram of the hepatoprotection of five kinds of flavonoids against liver injury induced by chronic alcohol consumption. Que, quercetin; Api, apigenin; Nar, naringenin; EGCG, epigallocatechin gallate; Gen, genistein.

We compared the effect of five structurally dissimilar flavonoid subgroups on alcoholic liver injury and discussed the probable mechanisms. The summary of how these flavonoids alleviated the hepatic injury induced by chronic alcohol consumption is shown in Figure 8. Flavonol (quercetin) had a beneficial function by inhibiting lipid peroxidation and oxidative stress (HO-1 activity). Flavone (apigenin) was efficient at improving hepatic function (ALT activity), serum dyslipidemia (LDL-C and TC levels in serum), lipid peroxidation and oxidative stress (SOD and GSH-Px activities). Flavanone (naringenin) was most effective at restoring hepatic function (ALT activity) and reducing dyslipidemia (serum and hepatic TC level), oxidative stress (CAT activity), and apoptosis. Flavanol (EGCG) provoked a significant improvement in serum dyslipidemia (HDL-C level), oxidative stress (GSH-Px activity), and inflammatory stress (MCP-1 and iNOS levels). Isoflavone (genistein) exerted benefits by attenuating serum dyslipidemia (TC level in serum), lipid peroxidation, oxidative stress (GSH activity), inflammatory stress (TNF-*α* level), and hepatic fibrosis. All the flavonoids could decrease serum and hepatic lipid accumulation (TG level), NF-*κ*B p65, COX-2, and IL-6 levels, and hepatic histopathological damage. These results can help us develop functional foods including one or more natural component(s) aiming to treat the liver injury induced by chronic alcohol consumption based on each action mechanism above. The structure‒function relationship needs to be investigated via the effect of different doses of other flavonoids on chronic alcohol-induced hepatic damage in future research.

## Figures and Tables

**Table 1:** Effect of five kinds of flavonoids on serum biochemical markers in mice ^1^.

Variables	NG	MG	Que	Api	Nar	EGCG	Gen
AST (U/L)	142 ± 12.29 ^a^	274 ± 21.94 ^f^	208 ± 13.13 ^e^	167 ± 10.68 ^bc^	195 ± 17.91 ^de^	182 ± 16.40 ^cd^	160 ± 13.15 ^b^
ALT (U/L)	30.0 ± 4.14 ^a^	56.0 ± 3.73 ^d^	40.3 ± 2.79 ^bc^	37.6 ± 2.56 ^b^	37.2 ± 1.85 ^b^	40.1 ± 1.93 ^bc^	42.6 ± 6.56 ^c^
HDL-C (mmol/L)	1.55 ± 0.12 ^d^	0.72 ± 0.08 ^a^	1.28 ± 0.24 ^b^	1.21 ± 0.12 ^b^	1.36 ± 0.14 ^bc^	1.53 ± 0.10 ^cd^	1.37 ± 0.16 ^bcd^
LDL-C (mmol/L)	0.976 ± 0.15 ^a^	2.12 ± 0.21 ^e^	1.43 ± 0.17 ^cd^	1.08 ± 0.12 ^ab^	1.45 ± 0.26 ^cd^	1.24 ± 0.17 ^bc^	1.55 ± 0.15 ^d^
serum TG (mmol/L)	1.24 ± 0.14 ^a^	1.81 ± 0.04 ^c^	1.57 ± 0.13 ^b^	1.42 ± 0.21 ^ab^	1.47 ± 0.24 ^b^	1.40 ± 0.17 ^ab^	1.55 ± 0.27 ^b^
serum TC (mmol/L)	3.39 ± 0.17 ^a^	5.05 ± 0.44 ^c^	4.75 ± 0.31 ^bc^	4.52 ± 0.22 ^b^	4.48 ± 0.20 ^b^	4.78 ± 0.34 ^bc^	4.58 ± 0.18 ^b^

^1^ Values represent the mean ± SD (*n* = 10). Labeled means without a common letter differ (*p* < 0.05). NG, normal group; MG, model group; Que, quercetin; Api, apigenin; Nar, naringenin; EGCG, epigallocatechin gallate; Gen, genistein; AST, aspartate aminotransferase activity; ALT, alanine aminotransferase activity; HDL-C, serum high-density lipoprotein cholesterol level; LDL-C, serum low-density lipoprotein-cholesterol level; TG, total triglyceride level; TC, total cholesterol level.
